# Integrative Oncology Fellowship Training in the United States: Uniting Requirements and Core Competencies

**DOI:** 10.1007/s13187-025-02643-w

**Published:** 2025-05-14

**Authors:** Ashley Larsen, Terri Crudup, Alissa Huston, Reya Sharman, Rodrick Babakhanlou, Renee Miu, Chirag Kapadia, Jean DiCarlo-Wagner, Tabarak Qassim, John Camoriano, Krisstina Gowin

**Affiliations:** 1 Cherng Family Center for Integrative Oncology, City of Hope, Orange County, CA USA; 2 Outcomes4Me, Philadelphia, PA USA; 3 Wilmont Cancer Institute, Pluta Integrative Oncology & Wellness Center, Rochester, NY USA; 4 Division of Hematology Oncology, University of Arizona, Tucson, AZ USA; 5 Society of Integrative Oncology, Washington, D.C USA; 6 Ipswich Hospital, East Suffolk and North Essex NHS Foundation Trust, Ipswich, UK; 7 Mayo Clinic, Phoenix, AZ USA; 8 City of Hope Comprehensive Cancer Center, Orange County, CA USA

**Keywords:** Integrative oncology, Integrative fellowship, Integrative medicine

## Abstract

Integrative oncology (IO) programs in cancer care institutions are increasing. However, specialized training in IO is still limited. In 2022, we launched the first Integrative Medicine in Hematology Oncology (IMHO) fellowship program in the United States. Here, we describe a roadmap and educational model for building a fellowship, utilizing the core competencies from Witt et al. and accreditation requirements of the Academic Consortium for Integrative Medicine and Health (ACIMH) as the foundation to build an IO fellowship program pilot program. The pilot program was based on a national needs assessment identifying the need for an integrative oncology training program. This review presents a theoretical framework for IO education. The framework is based upon the construction and successes of the IMHO fellowship pilot program. The fellowship’s foundation was built on the core competencies of integrative oncology knowledge, skill, and ability centered around the requirements of the ACIMH to meet requirement the of 1000 + hours in clinic, didactics, and research. Through the utilization of established integrative medicine curriculum married to integrative oncology-specific didactics, clinical application, and research initiatives, the pilot model has led to successful enrollment numbers, fellowship completion, and integrative medicine board eligibility. We put forward a roadmap for establishing IO educational programs, which can be tailored per center or institution. The IMHO pilot provides a clear and distinctive model to follow, allowing the ongoing need for fine-tuning and exploration of other models in the future to provide education across provider disciplines.

## Introduction

The advancement of cancer treatment has resulted in improved overall survival and life expectancy, and novel treatment approaches such as those offered with integrative medicine (IM). Integrative medicine focuses on the whole person, the patient-provider relationship, and is informed by evidence, encouraging all to use appropriate therapeutic and lifestyle approaches to achieve optimal health and healing [1]. Integrative oncology (IO), a more subspecialized form of IM, is formally defined as “patient-centered, evidence-informed, and uses mind and body practices, natural products, and lifestyle modifications from multiple cultural traditions alongside current cancer care treatments in order to optimize health, quality of life, and clinical outcomes” [2–4].

Cancer patients are increasingly interested in IO, with 60–80% of cancer patients reporting use [4, 5]. In fact, the majority of National Cancer Institute (NCI)-Designated Cancer Centers now offer IO services [4]. Despite this, there is a lack of trained IM providers to meet this exponential growth in demand [6]. This gap between demand for IO services and providers highlights the need for IM training programs to train providers, specifically in the area of IO. In order for IO and IM as a whole to flourish, there is a critical need for new providers and new provider models [7].

Education in IO is imperative to provide practitioners with the communication skills, competencies, and knowledge on guiding their patients in making decisions that marry their standard-of-care treatments to the IO practices [8]. It is crucial, when counseling on IO treatments, to ensure that the recommended IO therapies are evidence-based and differentiated from inadequate therapies when discussed with patients, in order to provide safe and effective treatment plans [8].

The demand for IO education in the United States (US) was evaluated in 2021, via a national survey-based needs assessment to gauge the interest in the development of an IO training program. The needs assessment surveyed 208 program directors, physicians, and fellows; 28% were “interested,” 17% were “highly interested,” and 22% were “very highly interested” in participating in an IO training program. The majority of the respondents felt IO was beneficial (37% agreed, 25% strongly agreed). In addition, 88% of respondents stated fellows should perform research as part of their IO education. Overall, IO training was perceived as valuable and desirable, and the development and dissemination of a training program with a research curriculum is needed [9].

Currently, very few formal training programs in IO exist in the US today; however, given strong national interest, several are in the development phase [6]. The rapid expansion of program interest and development highlights the necessity for a competent framework and national alignment for optimal delivery of training and education in IO. In this article, we review the previously outlined IO competencies, the path to board-eligibility in IM in the US, and suggest a potential framework for creation of IO training programs in the US.

## Methods

### Integrative Oncology Competencies

The educational competencies for IO as defined by Witt et al. [10] were reviewed. Witt et al. performed a systematic literature review on published competencies, and the results informed an international and interprofessional consensus procedure. The second phase consisted of three rounds of consensus procedure and included 28 experts representing 7 different professions (medical doctors, psychologists, nurses, naturopathic doctors, traditional Chinese medicine practitioners, yoga practitioners, patient navigators) as well as patient advocates, public health experts, and members of the Society for Integrative Oncology (SIO).

### Graduate Medical Education

We reviewed the policies and framework for Accreditation Council for Graduate Medical Education (ACGME) in Hematology Oncology. The ACGME is an independent, 501(c)(3), not-for-profit organization that sets and monitors voluntary professional educational standards essential in preparing physicians to deliver safe, high-quality medical care to all Americans. Graduate medical education (GME) refers to the period of education in a particular specialty (residency) or subspecialty (fellowship) following medical school; the ACGME oversees the accreditation of residency and fellowship programs in the US [11].

### Requirements for Board Eligibility in Integrative Medicine

Training programs for IO would optimally lead to eligibility for board certification in IM by the American Board of Physician Specialties (ABPS). The requirements for board-certification were reviewed according to the ABPS and The Academic Consortium of Integrative Medicine and Health (ACIMH) [12] program recognition requirements, both requirements needing to be filled for physician board-eligibility in IM.

### Sources of General Integrative Medicine Training

A comprehensive foundation in IM knowledge is required for both the broad education and foundation in IO, as well as board eligibility. As such, sources of general foundational IM were sought through educational partners, with printed and online sources investigated.

### Integrative Oncology Program Needs and Barriers Assessment

We reviewed the results of a previously conducted national needs assessment. This was an 18-question survey developed and sent to hematology/oncology, radiation oncology, and palliative care program directors, physicians, fellows, and internal medicine residents planning to enter any of the above fellowship program areas. Program directors and fellowship programs in all groups were identified through the ACGME. The distribution of the survey was completed on a national level utilizing Qualtrics to create and send the survey weekly in two 4-week sessions to all groups [13].

The survey included questions on basic demographic, and participant opinion, their experience and education, and their specialty training in the area of IO. In addition, participants were asked to provide their opinion on the barriers to an IO program, the type of education program they would like to see, and the topics they viewed as most relevant and/or beneficial to the field of IO. The survey contained structure questions, Likert-type opinion questions, and 1 to 10 rating scales.

### Integrative Oncology Training Program Pilot: A Use Case Study

In 2022, a feasibility and accessibility educational model was tested at the University of Arizona (UA) and Mayo Clinic Arizona (MCA) using the results of IO competency assessment and board-eligibility requirements for IM to inform the design of a dedicated IO fellowship.

## Results

### The Integrative Oncology Core Competencies

**Fig. 1:**
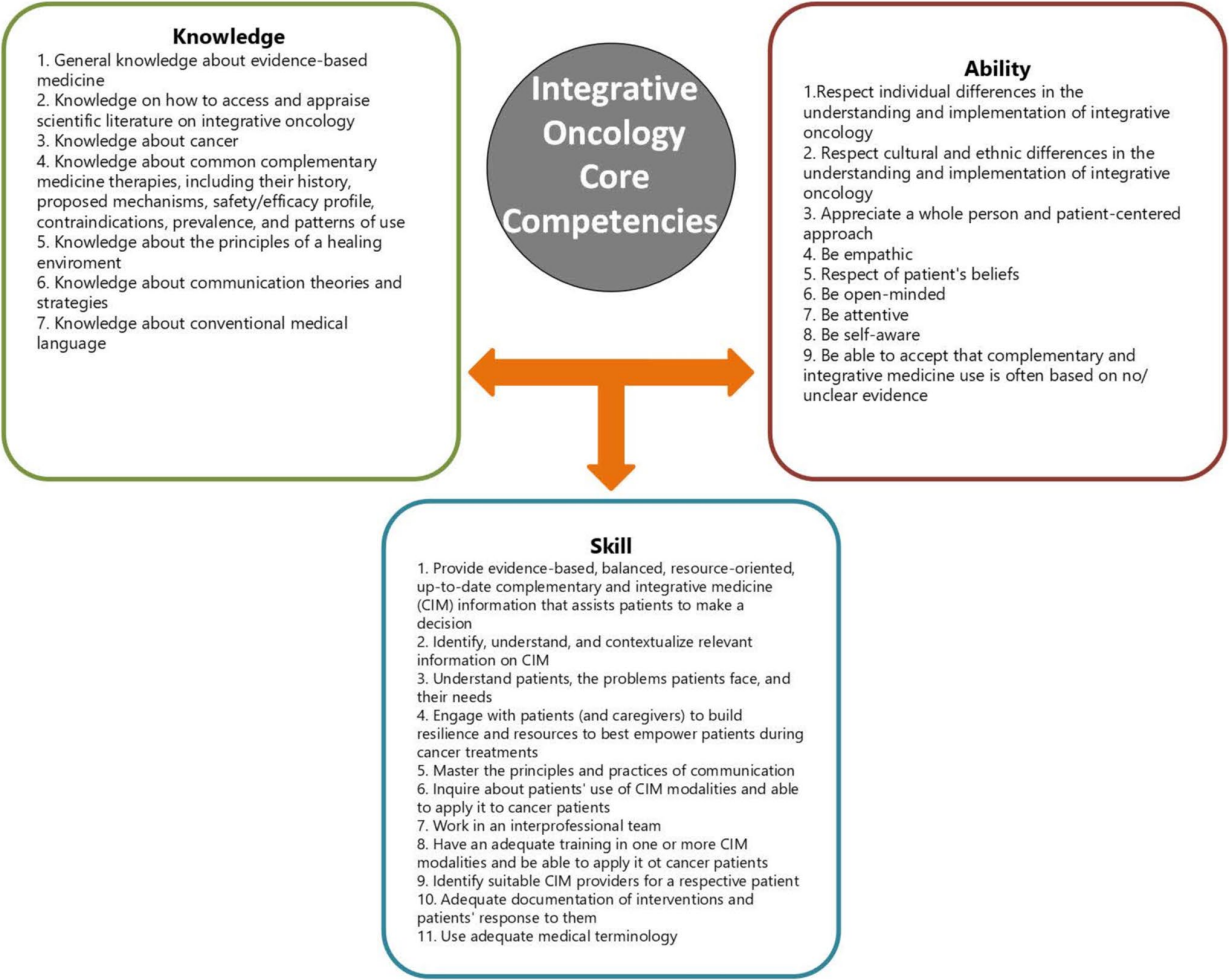
Integrative oncology core competencies.^10^

In the literature review performed by Witt et al., a total of 40 IM competencies were identified, and were further complemented by 18 core oncology competencies. The final round of the consensus procedure yielded 37 core competencies in the following categories: knowledge (*n* = 11), skills (*n* = 17), and abilities (*n* = 9). There was an agreement that these IO competencies are relevant for all participating professions [10] (Fig. 1).

### The Path to Board Eligibility in Integrative Medicine

In 2020, the ACIMH assumed the role of recognizing IM fellowship programs from the American Board of Physician Specialties. In 2023, the ACIMH modified their application for fellowship recognition to allow for specialty focused programs, such as in IO, outside of general IM to also receive recognition. Utilizing the framework of IM core competencies set forth by Ring et al. [14], the ACIMH sets and monitors the educational standard of IM fellowship programs. The core competencies in the ACIMH application are broken down into the following 6 main categories, with a classification of IM, Clinical Integration, and/or Research/Scholarly: Patient Care, Medical Knowledge, Practice Based Learning and Improvement, Interpersonal and Communication Skills, Professionalism, and System Based Practice.

The ACIMH breaks down the delivery method requirements of the above main categories further into minimum hours of total training, all adding up to a minimum of 1000 h total. A minimum of 400 h are required to be devoted to core/general IM education. This education needs to be a blend of different learning activities which are completed synchronously and asynchronously in the area of didactics, in-person, online, experiential, and/or in any combination. Secondly, a minimum of 100 clinical integration hours are required. The clinical hours should include 2 weeks of in-person and/or telemedicine time and 20 h of case study discussions.

A maximum of 200 h of IO research and/or scholarly projects is acceptable to be applied towards the 1000 h requirement. These types of activities can include but are not limited to preparation and presenting of lectures, abstracts or articles, education research, peer reviewed grant applications, education in research, and conducting or participating with other faculty on a clinical research trial. The remaining 300 minimum hours can be fellow focused training, such as participation in sub-specialty education, and it is highly suggested that these hours be applied in the core/general IM and/or clinical areas of training.

### Requirements for Board Eligibility in Integrative Medicine

For a fellow to be board eligible for the ABPS Board of Integrative Medicine (ABOIM), they must meet the required IM training and experience requirements. The three primary requirements are (1) their residency training must be from an approved program, such as the ACGME/American Osteopathic Association, (2) all applicants must currently hold, or have had, a board certification, such as from American Board of Internal Medicine or ABPS, and (3) they must complete a ACIMH approved fellowship in IM. Lastly, to complete the application to sit for ABOIM, three letters of recommendation are needed from board-certified diplomates, a self-query in the National Practitioner Data Bank report, and a current curriculum vitae must be submitted.

### Sources of General Integrative Medicine Curriculum

As there is no specific set of IO specific curriculum at this time, the following established sources of generalized IM curriculum, IO specific texts, and established society guidelines and references can be used as a foundation starting point for IO training:

*Andrew Weil Center for Integrative Medicine (AWCIM) Fellowship Foundational Curriculum *[15]* (FCC)*: This curriculum is an ACIMH approved curriculum and is divided into 13 units, providing topics including, but are not limited to introduction to IM, nutritional health, botanicals and dietary supplements, mind–body medicine, traditional whole systems of healing, and integrative approaches across major clinical specialties. This curriculum is applied to the study of IM as a whole and is not specifically focused on the subspecialty of IO.

*Integrative Medicine, 5 th Edition Rakel *[16]: This textbook is used as the standard for IM and was written by physicians who are experts in the field of IM. The textbook is broken down into a clinical, disease-oriented approach. The text describes incorporating IM therapies into clinical care and covers topics included, but not limited to: botanicals, supplements, mind–body, lifestyle choices, nutrition, exercise, and spirituality.

*Integrative Oncology, 3rd edition Abrams & Weil *[17]: This text is used to outline and review the basic principles of IO broken down by modalities and common cancer types. The text includes case histories, clinical pearls, and tables to bring attention to key and quick reference information.

*Society for Integrative Oncology (http//integrativeonc.org)*: The SIO is a non-profit organization which allows for practitioners from all levels and practices to come together internationally to provide communication, education, and research on IO. The goal of this professional society is to bring together evidence-based advocacy and research for the use of integrative care alongside standard of care treatment for cancer patients. Members of this society represent a vast majority of multidisciplinary practices from modern medicine to traditional medicine and other IM practices, in addition to patients and patient advocates. Additionally, the SIO has published several provider guidelines independently and in collaboration with the American Society of Clinical Oncology (ASCO) and has created educational modules for continuing medical education credit (https://integrativeonc.org/training-modules/) [18, 19].

### Integrative Oncology Program Needs and Barriers Assessment

In the 2021 national needs assessment conducted by the UA and MCA, the survey was conducted to gauge interest and investigate barriers to the development of an IO specific training program [9]. The survey consisted of a consent and 18-questions to be completed online. The survey was sent out to hematology/oncology, radiation oncology, and palliative care directors, physicians, fellows, and internal medicine residents planning to enter any of the above fellowship programs. The program directors and fellowship program were identified through the ACGME.

Participants (*n* = 208) included physicians from: hematology/oncology 65.9% (*n* = 137), radiation oncology 8.6% (*n* = 18), and palliative care 25.4% (*n* = 53). Hematology oncology respondents included physicians 47.4% (*n* = 25), fellows 33.6% (*n* = 46), and residents planning to enter into a hematology oncology fellowship 3.6% (*n* = 5). Program directors (*n* = 69) from each specialty included 15.3% (*n* = 21) from hematology/oncology, 61.1% (*n* = 11) from radiation oncology, and 69.8% (*n* = 37) from palliative care. Interestingly, palliative care had the highest response rate in this category. Survey respondents were mostly aged 31–40 years (38.4%), 41–50 years (23.8%), or 51–60 years (21.5%), with participant genders split by male (45.7%), female (50.9%), or prefer not to identify (3.5%). There was diverse regional participation across the United States. Overall participant responses revealed significant interest for an Integrative Hematology Oncology training program, despite little (36.0%) to no (49.4%) previous training in IM.

When surveyed on curriculum topics, participants were most interested in, the following were selected to be of greatest interest: physician/provider wellness, stress reduction techniques, off-label use of medication, exercise/physical activity, nutrition, and lifestyle counseling. Survey participants stated preferred education delivery of topics would be via didactic lectures and clinical practice exposure. Lastly, survey participants were asked to identify the barriers to implementing an IO training program. Barriers identified included a lack of an established curriculum and inadequate budget/time/faculty experience to implement the program. Despite the barriers, a majority of participants (64%) expressed interest and a need for an IO training program.

### Integrative Medicine in Hematology Oncology (IMHO) Fellowship—Pilot Model

In July 2022, the UA and MCA collaborated on a pilot model for an IO fellowship, named the IM in Hematology Oncology (IMHO) Fellowship, and represent the first formal IO fellowship model within an ACGME hematology oncology fellowship in the United States [20]. Recruitment for the IMHO fellowship occurred after the UA and MCA Hematology Oncology traditional ACGME and National Residency Match Program (NRMP) match process. After fellows were successfully matched with the Hematology Oncology fellowship programs, fellows who expressed interest in the IMHO fellowship receive supplemental application materials (i.e., personal statement on interest in IO. Applicants were selected on strength of interest in IO, potential for contribution, and commitment to the field as estimated by application materials and personal interview, with a recruitment of 1 fellow at each academic center per year.

As the IMHO fellowship runs concurrently with the 3-year Hematology Oncology fellowship, the program education model is based on a 3-year program. After 3 years, IMHO fellows are board eligible in Hematology, Oncology, and IM. The program aligns with the ACIMH educational requirement guidelines and received ACIMH accreditation in May 2024. Following the ACIMH, the educational model for the IMHO is structured into three main components all building and interconnecting with one another: Clinical Training, Research, and Didactics. IMHO fellows receive of a minimum of 1000 h of additional training that are aligned with the 37 core IO competencies categorized into knowledge, skills, and abilities [10].

*Core curriculum*: Core competency in general IM is achieved by IMHO fellows completion of the AWCIM FFC. The FFC is 500 h of online-based learning formulated by the AWCIM to meet the ABIOM requirements to sit for the ABIOM board certification exam.

As the FFC covers general IM, additional didactic training is required to specifically address the area of IO. Fellows are required to complete a series of independently curated curriculum specifically applicable to IO topics. Fellows participate in web-based educational videos, activities, and quizzes in diverse IO topics. Additionally, fellows are required to participate in scholarly activities, such as delivering lectures and presentations to students and other health professionals on IO-related topics.

The clinical training is accomplished through the practical application of IO components and modalities in a supervised clinical setting. The fellow is evaluated by a physician trained in IO practices in designated clinics. A fellow must demonstrate the following competencies: (1) coordinating and leading patient and caregiver visits and discussions on appropriate IO practices, as applicable to the individual patient, (2) practicing empathic and motivational interviewing techniques and ability to actively listen, (3) demonstrating the application of IO modalities to individual patients, (4) providing expert guidance for coordination of IO care, and (5) incorporating the patient’s cultural, racial, spiritual, and ethical preferences into their care.

In the IMHO Pilot model, fellows are required to complete an investigator-initiated clinical research project focusing on a component of IO. Fellows are matched with a mentor with a history of research experience. The clinical research projects are extended over the 3 years of training:

1. PGY4 Training Year: Fellows in their first year are provided with intense didactics on basic research skills and training, including CITI, HIPAA, and clinical trial design. Program directors at each site will match a fellow to the appropriate mentor that reflects the fellow’s area of research interest.
2. PGY-5 Training Year: Fellows in their second year continue with ongoing research didactics with focus on implementation and dissemination skills, project management, and ongoing team mentorship.
3. PGY-6 Training Year: Fellows in their third and final year continue with ongoing research didactics with focus on data analysis, scientific writing, and presentation skills.

### The Roadmap for Integrative Oncology Training Success

**Fig. 2:**
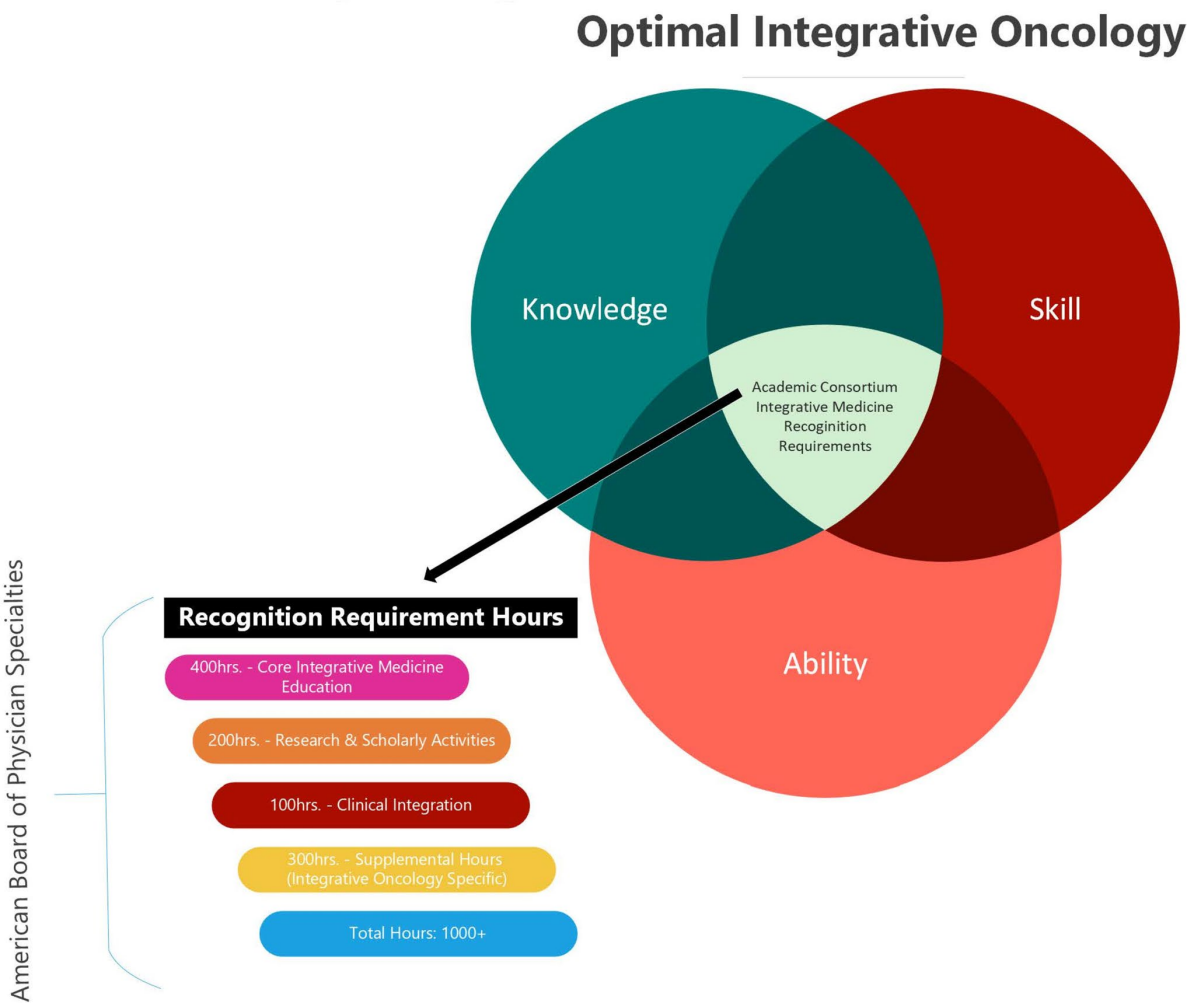
Theoretical framework for integrative oncology education

A successful IO fellowship program needs to follow the map outlined by the requirements set forth through ACIHM, ABIOM, and include incorporation of the core competencies of knowledge, skill, and ability (Fig. 2). These requirements set the foundation for a well-rounded, competent, and board eligible fellow at the end of their IO fellowship. These take into consideration the need for a balanced mixture of medical training, compassionate integrative care, and an evidence-based approach to providing IO consultations to patients.

The IMHO Fellowship took into consideration the ACIHM, ABIOM, and core competencies in creating a 3-year concurrent IO fellowship program to run alongside a hematology oncology fellowship [21]. The model has proven to be successful based on enrollment numbers, fellowship completion, ABIOM eligibility, and the receipt of ACIMH accreditation in 2024.

## Discussion

Cancer care centers and academic institutions around the United States increasingly recognize the importance of IO services, reflected in the worldwide growth in interest and utilization of this approach, especially by patients [2]. With increasing demand for IO modalities, a critical gap is emerging between the desire for services and ability to deliver IO expertise due to a lack of trained providers. This demand and service delivery gap has not gone unnoticed in the medical system by providers, and the majority surveyed agree education in IO is needed [9]. National standards and IO educational platforms are needed to meet this critical demand and ensure safe and appropriate utilization of IO services.

While general IM education programs have drastically increased in number [22], there are very few training programs in IM to meet sub-specialty needs, such as in IO. The current IM fellowship training program landscape is structured as an umbrella curriculum for general IM. There is a critical lack of depth in sub-specialty areas of focus, such as in oncology. Though certain parts of IM curriculum can be applied to multiple specialties, such as the topics of physical activity and nutrition, there is still a need for a specialty focused curriculum address these topics. IO textbooks and professional societies seek to fill this gap in the educational model, but more formal educational curriculum is desperately needed.

Institutions with interest in building IO training programs need to commence with the simultaneous understanding of the clinical competencies in IO practice within the domains of knowledge, skill, and ability, and the requirements to obtain board certification in the US. The conceptual overlay is intended to provide the theoretical framework of IO education for programs interested in developing novel IO educational models, but allow flexibility in programmatic design. This may serve as roadmap for institutions interested in programmatic development for IO. Additionally, the resources outlined to support the theoretical framework, such as the AWCIM FFC and integrative texts, may offer key content to assist in robust IO program curricular development. Finally, the IMHO pilot model can be considered as a potential structure for US-based ACGME hematology oncology programs interested in curating a unique track for rigorous training in IO.

The theoretical framework of IO outlines a potential roadmap to success. Institutions undertaking program development in IO are advised to first ensure the core general IM education content areas meet the requirements by the ACIMH and are delivered, either through independent content development or through licensing from other general IM educational platforms. Secondly, allocation of the minimum 1000 h over the proposed training period, as outlined by ACIMH, is critical. Thenceforth, the unique content pertinent to the sub-specialty should be curated and partnered with a strategy of curriculum delivery and competency assessment, which should align with the core competencies outlined under the three areas of focus: knowledge, ability, and skill [10]. Each of these core competencies need to then be married to the specific areas of patient care, medical knowledge, practice based learning and improvement, interpersonal skills, communication skills, professionalism, and systems based practices [14]. Finally, research and scholarly project emphasis should be weighed in the context of curriculum design and assessment.

The IMHO pilot model demonstrated the feasibility and acceptability of a 3-year concurrently run IO fellowship program [21]. While the success of this program directly informs the feasibility of such models for US-based ACGME hematology oncology programs, it concurrently highlights the need for additional types of program models. Feedback from the IMHO fellows [20], individuals inquiring about the program, and information gathered from the IO needs survey, all reveal other models of IO education are necessary to meet the needs of the candidate pool. Additional program models desired include a PGY-7 dedicated year track, a strictly clinical or research track depending on the fellow’s primary area of interest, faculty and established provider track, and programs for other healthcare providers (e.g., nurse practitioners, physician assistants, and nurse navigators). While this paper is focused on training models for physicians, other pathways along the spectrum of physician training and all members of the care delivery team should be considered as we address the educational needs for IO training.

As with any new program, there are challenges to development, dissemination, and sustainability. One of the primary challenges to creating a formal IO educational program is financial. Fellowship operations can be expensive and variable, depending on program size and the overall goals of the program [23, 24]. Expenses may include administrative and support staff, funding for curriculum content creation and platform hosting, textbooks, and more. Additional costs may include support for research projects, such as investigator seed funding and mentorship incentive. For the IMHO Pilot Program, funding for the fellowship came primarily through philanthropic gifts and grants. Though these are common sources of funding, they are not always sustainable for long-term. As such, financial sustainability models need to be explored, such as funding generated through an income stream generated from the clinical activity, fellow self-pay, fellowship endowments, or other institutional support.

Another important aspect of sustainability includes IO cultural buy-in from the institution hosting the fellowship. Institutional support is needed to support the multi-disciplinary approach to IO and to meet the clinical demand that emerges with increased focus on IO. There likely will be the need for additional resources and staff to implement services, and/or offer referrals to community integrative providers when housing an IO fellowship. Without the buy-in and preparedness for IO from the institution, an IO fellowship and the practice of IO will have barriers to success.

Implementation of a new training program will always be associated with barriers. The principal way of meeting these barriers will be through comprehensive preparation, careful planning, continual reassessment of successes and failures, collaboration and information sharing across institutions, and aligning research and outcomes to each IO education platform delivery.

## Conclusion

The roadmap provided in this paper, informed by resources, requirements, and core competencies can establish a firm foundation for IO education program development, which can be further tailored to meet the individual needs of a center or institution. The IMHO Pilot Model is a potential model for training programs to emulate for institutions striving to implement a successful training program within US-based hematology oncology training programs. However, exploration of other models of IO education are needed to address the spectrum of providers in practice across multiple disciplines. Finally, ongoing research is needed for optimal IO program development, implementation, and dissemination.

## Data Availability

Data is available upon request of the authors.
